# *Populus SVL* Acts in Leaves to Modulate the Timing of Growth Cessation and Bud Set

**DOI:** 10.3389/fpls.2022.823019

**Published:** 2022-02-17

**Authors:** Domenique André, José Alfredo Zambrano, Bo Zhang, Keh Chien Lee, Mark Rühl, Alice Marcon, Ove Nilsson

**Affiliations:** Department of Forest Genetics and Plant Physiology, Umeå Plant Science Centre, Swedish University of Agricultural Sciences, Umeå, Sweden

**Keywords:** poplar, FLOWERING LOCUS T, phenology, dormancy, SHORT VEGETATIVE PHASE

## Abstract

*SHORT VEGETATIVE PHASE (SVP)* is an important regulator of *FLOWERING LOCUS T (FT)* in the thermosensory pathway of Arabidopsis. It is a negative regulator of flowering and represses *FT* transcription. In poplar trees, *FT2* is central for the photoperiodic control of growth cessation, which also requires the decrease of bioactive gibberellins (GAs). In angiosperm trees, genes similar to *SVP*, sometimes named *DORMANCY-ASSOCIATED MADS-BOX* genes, control temperature-mediated bud dormancy. Here we show that *SVL*, an *SVP* ortholog in aspen trees, besides its role in controlling dormancy through its expression in buds, is also contributing to the regulation of short day induced growth cessation and bud set through its expression in leaves. *SVL* is upregulated during short days in leaves and binds to the *FT2* promoter to repress its transcription. It furthermore decreases the amount of active GAs, whose downregulation is essential for growth cessation, by repressing the transcription of *GA20 oxidase*. Finally, the SVL protein is more stable in colder temperatures, thus integrating the temperature signal into the response. We conclude that the molecular function of *SVL* in the photoperiodic pathway has been conserved between Arabidopsis and poplar trees, albeit the physiological process it controls has changed. SVL is thus both involved in regulating the photoperiod response in leaves, modulating the timing of growth cessation and bud set, and in the subsequent temperature regulation of dormancy in the buds.

## Introduction

Photoperiod is an important environmental cue that controls diverse developmental processes in plants, for example, flowering in Arabidopsis and timing of growth cessation in *Populus* trees (Pin and Nilsson, 2012). At the center of the mechanism, with which plants sense day length, is the *CONSTANS*/*FLOWERING LOCUS T* module. This module is partially conserved between *Populus* and Arabidopsis, but best understood in the latter. In Arabidopsis, *FT* expression is tightly regulated by many factors and becomes a hub for the integration of different signals, which fine-tunes the response. In addition to photoperiod (Kobayashi et al., 1999), it is regulated by age (Wang, 2014), vernalization (Searle et al., 2006), and ambient temperature (Lee et al., 2007). *SHORT VEGETATIVE PHASE* (*SVP*) is part of the latter pathway and represses *FT* expression by binding to its promoter (Lee et al., 2007). In *Populus* two *FT* orthologs have been identified, called *FT1* and *FT2*. Only *FT2* has a comparable expression pattern to the Arabidopsis *FT*, being expressed in leaves under long photoperiods, while *FT1* is only expressed in buds during winter (Hsu et al., 2011).

For trees in boreal forests, fine-tuning of the photoperiod response is critical for survival; they need to adapt to the rapidly changing seasons. Especially during the autumn months, temperature and day length are decreasing quickly. Once the day length falls under the critical day length, a threshold for growth permitting conditions, the trees stop their growth and set terminal buds, which protect the enclosed leaf primordia and shoot apical meristems from the subsequent low temperatures (Rohde and Bhalerao, 2007). These short days (SDs) are a reliable signal, with which the trees can anticipate the onset of winter. The signal is transmitted through *FT2*, which is downregulated within a few days after shifts to SDs (Böhlenius et al., 2006; Hsu et al., 2006). Trees failing to downregulate *FT2* are unable to respond to the SD signal and continue growth indefinitely, while plants with reduced *FT2* expression respond more quickly (Böhlenius et al., 2006), leading to early growth cessation and bud set.

*CO* and *GI* have been identified as positive regulators of *FT2* in long days (LDs). However, their expression profiles do not dramatically change upon shift to SDs (Ding et al., 2018). Arabidopsis CO is rapidly degraded in the dark, thus unable to induce *FT* in SDs (Valverde et al., 2004), and it is so far unclear if the same is true for poplar CO. However, the lack of induction by CO is not enough to explain the rapid downregulation of *FT2* in SDs, especially since *GI* is still expressed and of higher relative importance for *FT2* expression (Ding et al., 2018). GI might contribute to the release of repressive activity on *FT2* expression, as has been shown for poplar CYCLING DOF FACORS (Ding et al., 2018). Such repressors might therefore contribute to the downregulation of *FT2* expression in response to shorter photoperiods. Another possible candidate for such a repressor would be *SVP*, a MADS domain-containing gene and a strong repressor of *FT* expression in Arabidopsis (Hartmann et al., 2000).

*SVP* homologs have been found in other tree species. For example, in peach trees, six *DAM* (dormancy-associated MADS-box) genes have been associated with the non-dormant phenotype of the *evergrowing* mutant (Bielenberg et al., 2008). *DAM1* and *DAM4* peak in their expression at the end of summer and are hypothesized to be involved in the regulation of growth cessation (Li et al., 2009). Also, in apple, *DAM*- and *SVP*-like genes have been suggested to control bud set and dormancy (Wu et al., 2017, 2021; Falavigna et al., 2019, 2021; Moser et al., 2020). Recently, a *Populus SVP* ortholog named *SVL* has been shown to be expressed in buds where it is involved in dormancy establishment and maintenance (Singh et al., 2018, 2019). However, all analysis so far has been focused on the role of *SVP*/*DAM* genes in the buds, and their role in regulating the photoperiodic response in leaves is still unclear.

Besides *FT2*, another important factor of the short-day response is gibberellins (GAs, Eriksson et al., 2000). GAs are growth-promoting hormones and work both through and independently of *FT2* (Eriksson et al., 2015). A decrease in the levels of active GAs is essential for growth cessation and bud set (Eriksson et al., 2000). So far it is poorly understood how the levels of active GAs are regulated upon shift to SD. In short-day grown Arabidopsis, SVP represses the expression of *GA20 oxidase*, a gene encoding a rate-limiting enzyme in the GA biosynthesis pathway, thereby keeping the amount of bioactive GAs low (Rieu et al., 2008; Andrés et al., 2014). If this function of *SVP* was conserved in trees, it could be another mechanism through which *SVL* could potentially control growth cessation.

The ability of *SVP* to control *FT* expression and GA biosynthesis in Arabidopsis as well as the involvement of *MADS* genes in the phenology of other tree species prompted us to investigate the role of *SVL* in the regulation of growth cessation and bud set in *Populus*. Our data show that *SVL* expression in the leaves modulates the timing of SD-induced growth cessation and is able to repress both *FT2* and *GA20 oxidase* by binding to their promoters. Thus, *SVPs* mode of action has been conserved between Arabidopsis and *Populus*, even though the biological process it is involved in has changed.

## Materials and Methods

### Plant Material and Growth Conditions

Hybrid aspen (*Populus tremula × tremuloides*) clone T89 was used as WT control and all genetic modifications were done in this background. Plants were cultivated on ½ Murashige and Skoog medium under sterile conditions for 4 weeks or until they had rooted (max. 8 weeks). After transfer to soil, plants were grown in growth chambers in LD (18 h light, 20°C/6 h dark, 18°C) and with weekly fertilization (10 ml NPK-Rika S/plant). To induce growth cessation, plants were moved to SD (14-h light, 20°C/ 10-h dark, 18°C) and fertilization was stopped. For dormancy release, plants were treated with cold (8 h light, 6°C/16 h dark, 6°C). In both SD and LD, previously published bud scores (Ibáñez et al., 2010) were used to assess effects on bud development (set/flush). For year-around gene expression analysis, a *ca.* 40-year-old local (Umeå, Sweden) aspen tree was sampled once a month around midday (May to August leaves, buds from September to April).

### Phylogenetic Analysis

Protein sequences of SVP homologues were aligned in CLC main workbench (Qiagen) and a Maximum Likelihood Phylogeny was constructed with neighbor-joining method and 1,000 bootstrap replicates.

### Cloning of Plasmids

To generate *SVL* RNAi plants, the RNAi fragment was amplified by PCR using PtSVLRNAiF and PtSVLRNAiR primers, which contain attB1 and attB2 sites, respectively. The fragment was introduced into the pDONOR 201 vector (Invitrogen) by BP recombination. The PttSVLRNAi fragment was then transferred to the final destination vector pK7GWIGWI (Karimi et al., 2002) with Invitrogen LR recombinase, creating a double-stranded RNAi molecule driven by the constitutive Cauliflower Mosaic Virus *35S* promoter. For construction of PttSVLoe—(*35S::PttSVL:Myc*), full-length *PttSVL* CDS was amplified from hybrid aspen mRNA with oxPttSVLF and oxPttSVLR primers and cloned into pDONOR 201 with BP clonase (Invitrogen). The fragment was then transferred to the destination vector pGWB18 (Karimi et al., 2002). Primers used for construct generation are listed in Supplementary Table S1. All cloning reactions were performed according to the manufacturer’s instructions. Hybrid aspen was transformed as previously described (Nilsson et al., 1992). *Arabidopsis thaliana* was transformed by using the floral dip method (Clough and Bent, 1998). The *svp-32* (Salk_072930) mutant seeds were ordered from Nottingham Arabidopsis Stock Centre (NASC).

### Analysis of *SVP*-Overexpressing Arabidopsis Plants

*Arabidopsis thaliana* WT Col-0, *svp-32,* and PttSVLoe plants were grown on soil in LD (16 h light/8 h dark, 22°C). To measure flowering time, rosette leaves and cauline leaves of 10 plants per line were counted until first flowers were visible.

### RNA Extraction and Quantitative Real-Time PCR

Poplar leaves (youngest fully expanded leaves) were ground to fine powder, of which 100 mg were used for RNA extraction with CTAB extraction buffer (Chang et al., 1993; 2% CTAB, 100 mM Tris-HCl (pH 8.0), 25 mM EDTA, 2 M NaCl, 2% PVP). The samples were incubated at 65°C for 2 min and extracted twice with an equal volume of chloroform–isoamyl alcohol (24:1). Nucleic acids were precipitated at −20°C for 3 h with ¼ volumes 10 M LiCl. Precipitate was collected by centrifugation (13,000 rpm, 4°C, 20 min) and washed with 70% EtOH. After drying it was dissolved in 60 μl H2O (DEPC treated). Contamination of genomic DNA was removed from 2,5 μg total nucleic acid by DNase treatment (TURBO DNA-free^™^ Kit, Ambion^®^), and 1,000 ng RNA were used for cDNA synthesis with iScript^™^ cDNA Synthesis Kit (Bio-Rad). The cDNA was diluted 50 times for downstream applications. Quantitative real-time PCR (qPCR) was run on a LightCycler^®^ 480 with SYBR Green I Master (Roche). All kits and machines were used according to the manufacturer’s instructions. The reaction protocol started with 5 min pre-incubation at 95°C, followed by 50 cycles of amplification consisting of 10 s denaturation at 95°C, 15 s annealing at 60°C and 20 s elongation at 72°C. For the acquisition of a melting curve, fluorescence was measured during the step-wise increase in temperature from 65°C to 97°C. Relative expression levels were obtained using the 2^-ΔΔCq^ method (Livak and Schmittgen, 2001). GeNorm (Vandesompele et al., 2002) identified UBQ and 18S as most stable reference genes. All used primers had an efficiency of >1,8 and their correct product was confirmed by sequencing. A complete list of primer sequences can be found in Supplementary Table S1.

### GA Quantification

Material (about 150 mg fresh weight of the youngest fully expanded leaves) was suspended in 80% methanol–1% acetic acid containing internal standards and mixed by shaking during 1 hour at 4°C. The extract was kept a − 20°C overnight and then centrifuged and the supernatant dried in a vacuum evaporator. The dry residue was dissolved in 1% acetic acid and passed through a Oasis HLB (reverse-phase) column as described in (Seo et al., 2011). The dried eluate was dissolved in 5% acetonitrile–1% acetic acid, and the GAs were separated using an autosampler and reverse-phase UHPLC chromatography (2.6 μm Accucore RP-MS column, 100 mm length x 2.1 mm i.d.; Thermo Fisher Scientific) with a 5 to 50% acetonitrile gradient containing 0.05% acetic acid, at 400 μl/min over 21 min.

The hormones were analyzed with a Q-Exactive mass spectrometer (Orbitrap detector; Thermo Fisher Scientific) by targeted Selected Ion Monitoring. The concentrations of GAs in the extracts were determined using embedded calibration curves and the Xcalibur 4.0 and TraceFinder 4.1 SP1 programs. The internal standards for quantification were the deuterium-labeled hormones.

### RNA Sequencing Analysis

For RNA sequencing experiments RNA was isolated as described above and purified with RNeasy kit (Qiagen) according to the manufacturer’s instructions. DNAse treatment was performed on column (Qiagen). Concentration and quality of RNA were assessed using Qubit^™^ RNA BR Assay Kit (Invitrogen) and Bioanalyzer (Agilent), respectively. 3 μg total RNA with RIN ≧8 were sent for sequencing to SciLife Lab, Stockholm. Library preparation was carried out with an Agilent NGS Bravo workstation in 96-well plates with TruSeq Stranded mRNA kit (Illumina) according to the manufacturer’s instructions. mRNA was purified through selective binding to poly dT-coated beads and fragmented using divalent cations under elevated temperature. cDNA was synthesized using SuperScript II Reverse Transcriptase (Thermo Fisher Scientific), cleaned with AMPure XP solution (Thermo Fisher Scientific), 3′ adenylated, and ligated to adapters. Fragments were cleaned with AMPure XP beads (Thermo Fisher Scientific), amplified by PCR, and purified with AMPure XP beads (Thermo Fisher Scientific). After washing with 80% ethanol, they were eluted in EB (Qiagen). The quality and concentration of the adapter-ligated libraries were checked on the LabChip GX/HT DNA high sensitivity kit and by Quant-iT, respectively. The libraries were then sequenced using the Illumina NovaSeq-6,000 platform, generating from 20 to 110 million paired-end reads (2 × 150 bp) per sample.

### Pre-processing of RNA-Seq Data and Differential Expression Analyses

The data pre-processing was performed as described here: http://www.epigenesys.eu/en/protocols/bio-informatics/1283-guidelines-for-rna-seq-data-analysis. The quality of the raw sequence data was assessed using FastQC.^1^ Residual ribosomal RNA (rRNA) contamination was assessed and filtered using SortMeRNA [v2.1 (Kopylova et al., 2012); settings--log --paired_in --fastx--sam --num_alignments 1] using the rRNA sequences provided with SortMeRNA (rfam-5 s-database-id98.fasta, rfam-5.8 s-database-id98.fasta, silva-arc-16 s-database-id95.fasta, silva-bac-16 s-database-id85.fasta, silva-euk-18 s-database-id95.fasta, silva-arc-23 s-database-id98.fasta, silva-bac-23 s-database-id98.fasta and silva-euk-28 s-database-id98.fasta). Data were then filtered to remove adapters and trimmed for quality using Trimmomatic [v0.39 (Bolger et al., 2014); settings TruSeq3-PE-2.fa:2:30:10 LEADING:3 SLIDINGWINDOW:5:20 MINLEN:50]. After both filtering steps, FastQC was run again to ensure that no technical artefacts were introduced. Filtered reads were pseudo-aligned to v1.1 of the *P. tremula* transcripts {retrieved from the PopGenIE resource (Sundell et al., 2015) using salmon [v1.1.0 (Patro et al., 2017)], with non-default parameters --gcBias--seqBias --validateMappings} against an index containing the *P. tremula* v1.1 genome sequence as decoy. Statistical analysis of single-gene differential expression between conditions was performed in R (v4.0.0; R Core Team 2020) using the Bioconductor [v3.10 (Huber et al., 2015)] DESeq2 package [v1.28.1 (Love et al., 2014)]. FDR adjusted values of *p* were used to assess significance; a common threshold of 1% was used throughout. For the data quality assessment (QA) and visualization, the read counts were normalized using a variance stabilizing transformation as implemented in DESeq2. The biological relevance of the data—for example, biological replicates similarity—was assessed by principal component analysis and other visualizations (e.g., heatmaps), using custom R scripts, available from https://github.com/DomeniqueA/SVL. The raw data are available from the European Nucleotide Archive^2^ under the accession number PRJEB46749.

### Protein Stability Assay

WT and SVLoe plants were grown in LD (18 h light, 20°C/6 h dark, 18°C) for 4 weeks before the experiment started. Upon shift to SD, half of the plants were transferred to regular SD (14 h light, 20°C/ 10 h dark, 18°C), while the other half were transferred to cold SD (14 h light, 15°C/10 h dark, 10°C). Proteins were isolated and visualized on a Western blot using anti-myc antibodies (Agrisera).

### Chromatin Immunoprecipitation Analysis

WT and SVPoe plants were grown in LD. Per genotype, one fully expanded leaf was harvested from each of four biological replicates at ZT 18 and cut into small pieces. These were cross-linked in 50 ml PBS buffer +1% formaldehyde and vacuum (4 times 5 min). The reaction was stopped with addition of glycine to a final concentration of 100 mM. The pieces of leaves were frozen in liquid N_2_ and ground to fine powder. Nuclei were extracted, lysed in nuclei isolation buffer [50 mM HEPES pH 7.4, 5 mM MgCl_2_, 25 mM NaCl, 5% sucrose, 30% glycerol, 0.25% Triton X-100, 0.1% ß-mercaptoethanol, 0.1% proteinase inhibitor cocktail (Sigma)] and sonicated, resulting in DNA fragments of 500–1,000 base pair length. For immunoprecipitation, 300 μl of the nuclear extract were homogenized with 200 μl IP buffer (80 mM Tris-HCl pH 7.4, 230 mM NaCl, 1.7% NP40, 0.17% DOC) followed by 1 μl 1 M DTT, 1 μl protease inhibitor cocktail, 1 μl 10 mg/ml RNase A and 5 μl of a monoclonal myc antibody (ab32, Abcam). The mixture was incubated under soft agitation at 4°C over night and centrifuged at full speed for 15 min at 4°C. 40 μl Protein A beads were added into the supernatant and incubation was continued for another 2 hours with soft agitation at 4°C. Protein beads were first washed two times with ice-cold low salt buffer (20 mM Tris-HCl pH 8, 150 mM NaCl, 0.1%SDS, 1% Triton X-100, and 2 mM EDTA), and two times with high salt buffer (20 mM Tris-HCl pH 8, 500 mM NaCl, 0.1%SDS, 1% Triton X-100, and 2 mM EDTA). Then, beads were washed two times with ice-cold LiCl buffer (10 mM Tris-HCl pH 8, 250 mM LiCl, 1% Igepal Ca-630, 1% DOC, and 1 mM EDTA). Chromatins were eluted from the beads with elution buffer (100 mM NaHCO_3_, 1% SDS) at 65°C for 20 min. To de-crosslink the extract, it was incubated with proteinase K (10 ng/ml) for 1 hour at 55°C. Afterward, DNA was extracted by Chromatin immunoprecipitation (ChIP) DNA Clean & Concentrator Kits (Biosite D5205). Quantities of immunoprecipitations were quantified using SYBR green (Roche) and the iQ5 light cycler (Bio-Rad). A similarly treated extract from WT without tagged protein was used as control. Primers used for amplification of genomic fragments are listed in Supplementary Table S1.

## Results

### *SVL* Is Functionally Similar to *AtSVP*

*Populus* has one orthologous gene to Arabidopsis *SVP* called *SVL* (Singh et al., 2018; Supplementary Figure S1A). *AtSVP* and *PtSVL* share 66% identity on the amino acid level, making *SVL* the only likely *SVP* ortholog compared to other MADS domain-containing genes in *Populus* (Supplementary Figure S1A; Singh et al., 2018). Because of the high similarity to *SVP* (Supplementary Figure S1B), we hypothesized that *SVL* could act like *SVP* in Arabidopsis by having a function in the photoperiodic response in leaves. The *svp* mutant is early flowering (Hartmann et al., 2000) and we tested whether *SVL* could rescue this phenotype. For this we expressed *SVL* cDNA under the control of the *35S* promoter in *svp-32* plants. Flowering time was determined by counting rosette and cauline leaves. These plants produced significantly more leaves than *svp-32* mutants and wild-type (WT) Arabidopsis plants before developing the first flowers (Supplementary Figure S2). These results imply that the functionality of has been conserved between Arabidopsis *SVP* and *Populus SVL*.

### Expression of *Populus SVL* Is Induced in Leaves During Short Days

**Figure 1:**
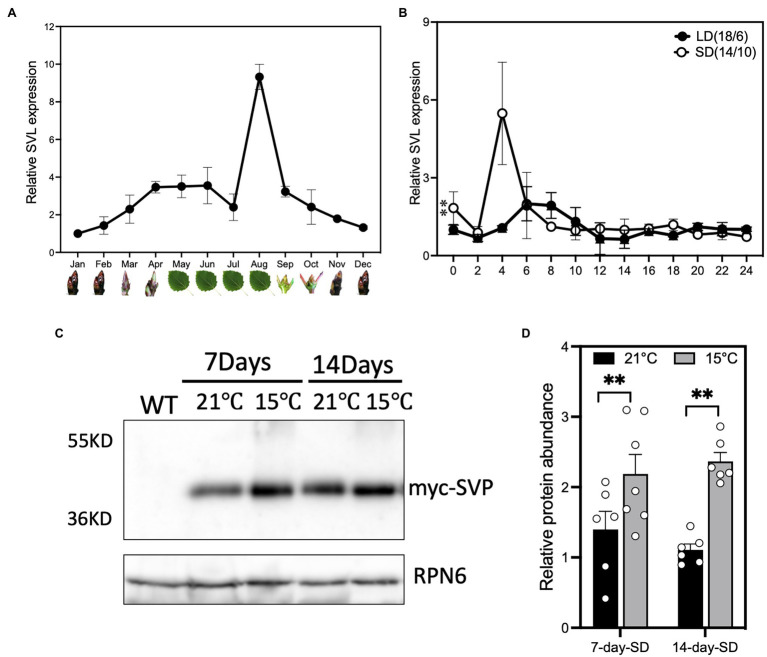
*SVL* is expressed during the autumn. **(A)** *SVL* expression in field-grown mature *Populus tremula* over the course of 1 year. Samples were taken at 2 p.m. in the middle of each month. May–August leaves, September–April terminal buds. Values are relative to the expression in January samples. Error bars indicate standard error of biological replicates, *n* = 3. **(B)** Relative *SVL* expression in WT during long days (18 h light/6 h dark) and after 2 weeks of short days (14-h light/10-h dark) treatment. Lowest expression in LD was set as 1. The asterisks indicate a statistically significant difference between LD and SD samples by two-way ANOVA Fisher’s test. Error bars indicate standard error of biological replicates, *n* = 3. ^**^Indicate *p* < 0.01. **(C)** Western blot showing that the myc-SVL protein accumulates in leaves of *35S::myc:SVL*-expressing plants during SD treatment at both 21°C and 15°C day temperature. **(D)** Relative protein abundance of myc-SVL in leaves of *35S::myc:SVL*-expressing plants after 1 week and 2 weeks of SD treatment at both 21°C and 15°C day temperature. All values are relative to the protein amount at 14-day SD at 21°C. The asterisks indicate a statistically significant difference between two temperature samples by Welch’s test. Error bars indicate standard error of biological replicates, *n* ≥ 6. ^**^Indicate *p* < 0.01.

*Populus SVL* function has previously been described in the shoot apex in relationship to the regulation of bud dormancy (Singh et al., 2018, 2019). We wanted to investigate to what extent leaf-expressed SVL also contributes to the regulation of growth cessation and bud set. Investigation of the *SVL* annual expression pattern in local adult aspen trees (Umeå, Sweden) sampled in the middle of the day showed that it is highly expressed in leaves during the short days (SDs) of late summer and early autumn (Figure 1A), after *FT2* expression declined (Supplementary Figure S3), and to higher levels than what can be detected in buds. To test whether this expression pattern is consistent in juvenile trees grown in controlled growth conditions, we checked the diurnal expression pattern of *SVL* in leaves first in long days (LD) and after 2 weeks of SD treatment (Figure 1B). In these conditions, one of the first genes to respond is *FT2* which shows a clear downregulation after 2 weeks in 14 h SD (Ding et al., 2018). In long days, *SVL* displayed a minor peak of expression at ZT 6–8 (Figure 1B). The *SVL* expression increased after shift to SD and showed a prominent morning peak at around 4 hours after dawn, suggesting a role for *SVL* in the photoperiodic response in leaves. We then also wanted to know if a decrease in ambient temperature could increase the stability of the SVL protein, as has been shown for Arabidopsis SVP (Lee et al., 2013). When plants expressing myc-tagged SVL from a constitutive promoter were exposed to lower temperatures than our standard SD treatment, the accumulation of SVL protein was increased (Figures 1C,D). This indicates that there could be a role for leaf-expressed *SVL* in response to both short photoperiods and lower temperatures.

### *SVL* Is Promoting SD Induced Growth Cessation

**Figure 2:**
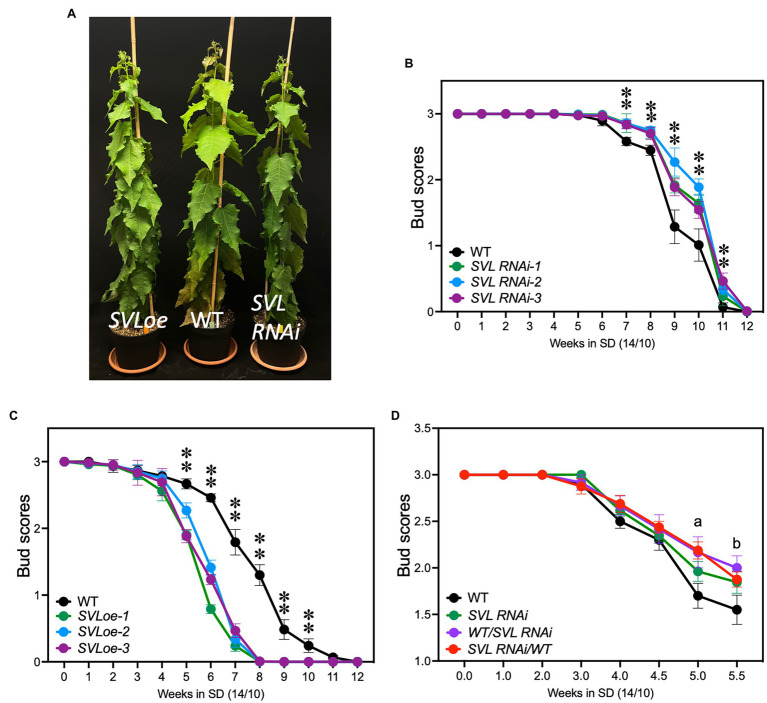
*SVL* controls growth cessation and bud set. **(A)** Photograph of SVLoe, WT, and *SVL* RNAi plants after 3 weeks in LD. **(B)** Plot of bud scores showing that growth cessation and bud set of three independent *SVL* RNAi lines were delayed compared to WT. Error bars indicate standard error of biological replicates, *n* = 9. **(C)** Plot of bud scores describing growth cessation and bud set of three independent SVLoe lines compared to WT. SVLoe plants stopped growth earlier than WT. The asterisks indicate statistically significant difference of each transgenic line from WT by two-way ANOVA Fisher’s test. Error bars indicate standard error of biological replicates, *n* = 9. ^**^Indicate *p* < 0.01. **(D)** Plot of bud scores showing growth cessation of WT, *SVL RNAi*, and grafts thereof. *a*, significant differences were observed from multiple comparisons of WT vs. *SVLRNAi*, WT vs. *WT/SVLRNAi*, WT vs. *SVLRNAi/WT*, and *SVLRNAi* vs. *SVLRNAi/WT*. *b*, significant differences were observed from multiple comparisons of WT vs. *SVLRNAi*, WT vs. *WT/SVLRNAi*, and WT vs. *SVLRNAi/WT.* Statistic significance was determined by two-way ANOVA Fisher’s test (*p* < 0.05). Error bars indicate standard error of biological replicates, *n* ≥ 6.

To test the role of *SVL*, we generated *SVL* RNAi and *SVL* over-expressing (*SVLoe*) trees. Downregulation was up to 80% effective, while overexpression resulted in a six-fold increase of *SVL* expression at ZT17 compared to wild-type T89 (WT; Supplementary Figure S4). Neither downregulation nor overexpression of *SVL* had a striking effect on vegetative growth; all transgenic lines were indistinguishable from WT controls after 3 weeks in LD (Figure 2A). After shift to SD, poplars respond with growth cessation and bud set. We used previously described bud scores (Ibáñez et al., 2010) to test the speed of SD response in three independent transgenic lines per construct. *SVL* RNAi plants showed a small but consistent delay of bud set compared to WT (Figure 2B). Both growth cessation (score 2) and bud set (score 1) were delayed by *ca.* 1 week. *SVL*oe plants on the other hand ceased growth several weeks earlier than WT (Figure 2C). This indicates that *SVL* is a repressor of vegetative growth and promoter of SD-induced growth cessation.

### *SVL* Acts in Both Leaf and Shoot Apex to Promote SD-Induced Growth Cessation

Expression of both *FT2* and *GA20oxidase* in rootstocks of grafted trees is sufficient to significantly delay growth cessation and bud set (Miskolczi et al., 2019). We then asked if the role of SVL in modulating the timing of growth cessation is due to SVL activity in the leaf or shoot apex or both. To investigate this we performed reciprocal graftings of *SVL* RNAi and wild-type trees and compared the timing of growth cessation to trees where scions had been grafted to their own stock. In both types of heterografts growth cessation was delayed to the same extent as in SVL RNAi homografts suggesting that SVL modulates the timing of growth cessation trough activity both in the leaf and in the shoot apex (Figure 2D).

### *SVL* Regulates Growth Cessation Through Repression of *FT2* Expression and Gibberellin Biosynthesis

**Figure 3:**
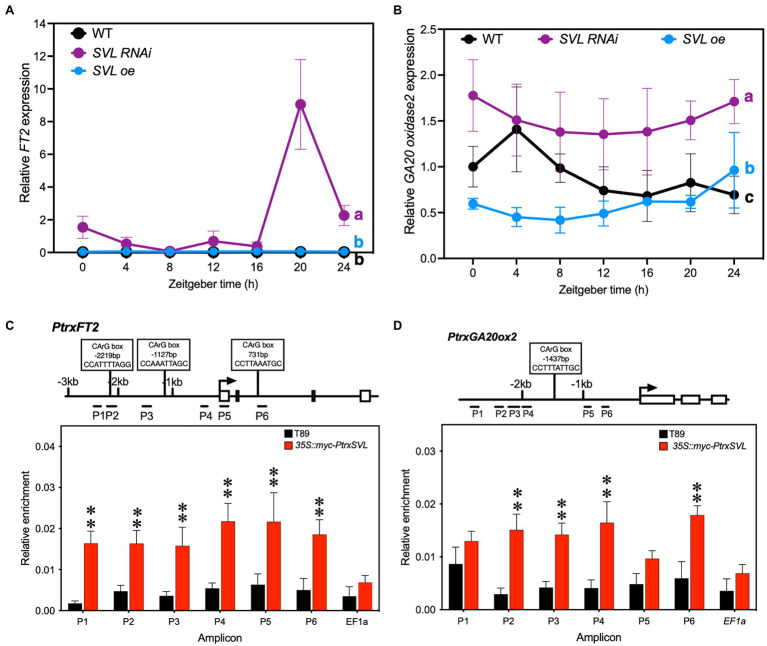
SVL is a transcriptional repressor and is associated with the promoters of its targets. **(A,B)** Gene expression of *FT2* **(A)** and *GA20 oxidase2* **(B)** after 2 weeks of SD (14 h light/10 h dark) treatment. In both cases expression was increased in *SVL* RNAi and decreased in SVLoe lines. Lines that do not share letters are significantly different from each other according to post-two-way ANOVA Fisher’s test (*p* < 0.05). Error bars indicate standard error of biological replicates, *n* = 3. **(C,D)** Relative enrichment of promoter fragments after ChIP quantified by qPCR of *FT2* **(C)** and *GA20 oxidase2* **(D)**. Values are normalized against input. Position of putative SVL binding sites, CArG boxes, shown to bind to Arabidopsis SVP in leaves (Gregis et al., 2013), are indicated. The asterisks indicate a statistically significant difference from WT by Welch’s test. Error bars indicate standard error of biological replicates, *n* = 4, ^**^indicate *p* < 0.01.

We then tested whether the different speeds of response in transgenic lines were due to altered expression of *FT2*. After 2 weeks of SD treatment, *FT2* expression had ceased in WT and *SVLoe*, while it was still strongly expressed in the *SVL* RNAi lines (Figure 3A). In addition to *FT2*, gibberellins are also known to affect growth cessation and bud set. We therefore analyzed the expression of a *GA20 oxidase2*, a key enzyme in gibberellin biosynthesis and found that it was increased in the leaves of *SVL* RNAi lines, while being reduced in SVLoe (Figure 3B). We focused on *GA20 oxidase2* because we have found that it is the predominantly expressed *GA20 oxidase* gene in leaves (not shown). Consequently, the amount of the active gibberellin GA_1_ was increased in leaves of *SVL* RNAi lines in both LD and SD (Supplementary Figure S5) compared to wild type. This suggests that SVL can influence the timing of growth cessation through a repression of both the expression of *FT2* and the biosynthesis of gibberellins.

### SVL Binds to the Promoters of Its Downstream Targets

We then asked if *FT2* and *GA20 oxidase2* are direct targets of SVL. Since *SVL* is a MADS-box transcription factor, we tested the ability of the SVL protein to bind to the promoter region of these genes. For that we performed a chromatin immunoprecipitation assay in leaves of WT and our myc-tagged SVLoe lines. Quantification by qPCR showed significant enrichments of six fragments surrounding the *FT2* transcriptional start site, up to 2.5 kb upstream and 1 kb downstream (Figure 3C). Enrichment at the *GA20 oxidase2* promoter was significant for four of six fragments (Figure 3D). No enrichment could be detected at a control locus (Figures 3C,D). These results show that SVL can associate with the promoters of *FT2* and *GA20 oxidase2* and potentially repress their expression through direct binding.

### SVL Has a Minor Influence on the Leaf Transcriptome

**Figure 4:**
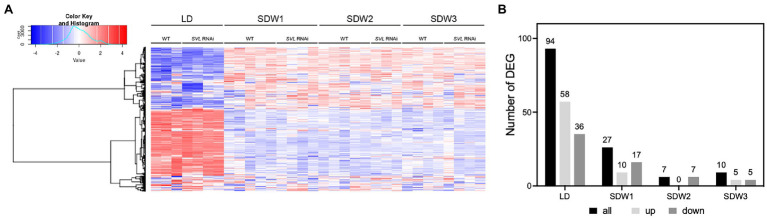
Global transcriptional changes in response to SD treatment. **(A)** Heatmap of gene expression in WT and *SVL* RNAi in LD and SD (week 1 to week 3). **(B)** Number of differentially expressed genes between WT and *SVL* RNAi over time.

To better understand what role SVL plays in leaves during SD treatment, we performed RNA sequencing analysis on leaves of wild-type and SVL RNAi plants. Samples were harvested at ZT17 during LD and after one, two, three and 10 weeks of SD treatment, respectively. Major transcriptional changes happened after the shift from LD to SD in both WT and *SVL* RNAi lines with more than 10,000 genes being differentially expressed between the two time points (Figure 4A; Supplementary Figure S6). However, no further significant changes were detectable after two and 3 weeks of SD. At the individual time points, only a small number of differentially expressed genes (DEG) could be detected between the two lines (Figure 4B; Supplementary Figure S6) and gene ontology (GO) enrichment resulted in no specific terms. These results indicate that the role of SVL in leaves might be limited to the regulation of a very limited set of genes.

## Discussion

### *AtSVP* and *PtSVL* Are Functionally Conserved and Repress *FT*

*AtSVP* is a known floral repressor (Hartmann et al., 2000), acting on the *FT* promoter (Lee et al., 2007). Here we show that *PtSVL* (hereafter *SVL*) is functionally conserved as was recently shown for apple (Falavigna et al., 2021) and previously also, for instance, Kiwi *SVP*-like genes, (Wu et al., 2012). Both SVP and SVL proteins share 66% identity and *SVL* overexpression was sufficient to complement the Arabidopsis *svp-32* mutant phenotype. ChIP analysis showed that SVL could bind to both *FT2* and *GA20 oxidase* promoters, repressing their transcription. This suggests that *SVL* expression in the leaf can fulfil a similar role in poplar as in Arabidopsis, modulating the photoperiodic regulation of *FT* and *GA20 oxidase* expression. To repress downstream targets, AtSVP forms heterodimers with MADS-box proteins like FLM and FLC (Lee et al., 2007, 2013; Posé et al., 2013). In apple, it has recently been shown that the SVP-like protein MdSVPa can form transcriptional complexes with various MADS-box proteins that are expressed in buds at different dormancy-specific phases (Falavigna et al., 2021). It remains to be shown if the SVL activity in the *Populus* leaf is also dependent on one or more MADS-box proteins.

### Cold Temperatures Promote SVL Activity

In Arabidopsis, *SVP* is part of the thermosensory pathway, which inhibits flowering in cold conditions (Lee et al., 2007), partially mediated by a stabilization of the SVP protein at colder temperatures (Lee et al., 2013). Temperatures drop during autumn and we could show that SVL protein was more abundant in a combination of short-day treatment and cold (Figure 1C), mimicking autumn conditions. While the timing of growth cessation is mainly regulated by photoperiod, integration of temperature signals through SVL could give trees more flexibility to fine-tune their SD response, it could also be an important factor contributing to SVLs function in buds during the induction of dormancy.

### *SVL* Regulates Growth Cessation Through *FT2* and GAs

There is no strict correlation between the downregulation of *FT2* and the upregulation of *SVL* in the year-around samples (Figure 1A; Supplementary Figure S3) confirming that *SVL* is not the primary regulator of the photoperiodic response and the regulation of *FT2*. However, alteration of *SVL* expression levels influenced the timing of growth cessation and bud set upon short-day (SD) treatment. We could show that this was caused by an effect on *FT2* expression and levels of GAs. Consistently, *SVL* RNAi lines had increased *FT2* expression and a delayed growth cessation, while SVLoe plants had strongly reduced *FT2* expression and early growth cessation. Furthermore, levels of the bioactive gibberellin GA_1_ were increased in *SVL* RNAi plants. This suggests that the role of *SVL* is to modulate the timing of the photoperiodic response.

The interest for the role of *SVP*-like genes in the regulation of dormancy originally stemmed from studies of the *evergrowing* mutant (*evg*) in peach (*Prunus persica*), which fails to form terminal vegetative buds and continues to grow indeterminately under dormancy-inducing conditions. The *evg* locus was mapped and found to contain a large deletion of six tandemly repeated *SVP*-like genes called *DAM* genes (Bielenberg et al., 2008). The simultaneous downregulation of the expression of three SVP/DAM genes in apple through RNAi also caused an evergrowing phenotype (Wu et al., 2021). Because of the focus on the expression and function of these genes during dormancy-induction in the bud, to our knowledge, there appears to be no characterization of the photoperiodic regulation of expression of *FT*-like genes in leaves of the evergrowing mutants. It is an interesting possibility that the loss of *SVP*/*DAM*-like expression in the leaves of peach and apple could contribute to an inability to keep *FT* expression down in response to the short-day signal. Since downregulation of *FT* expression appears to be a prerequisite for growth cessation and bud set (Böhlenius et al., 2006), this could at least partially explain the fact that the evergrowing mutants are not only failing to establish dormancy, but are also not able to enter into growth cessation and bud set—which is also a prerequisite for the entry into dormancy.

There are several other *SVP*-like and *DAM*-like genes in *Populus* (Supplementary Figure S1). In order to fully understand the role of the individual genes, it will be important to know if complete knockouts of *SVL* expression through CRISPR-Cas9-mediated gene editing, or combinations of the deactivation of several *SVP*/*DAM*-like genes, also leads to an evergrowing phenotype in poplar trees.

### SVL Regulates Growth Cessation and Bud Set in Both Leaves and Shoot Apices

Previous studies showed the role of *SVL* in the shoot apex, both during the establishment of dormancy during SD (Singh et al., 2019) and its release during winter (Singh et al., 2018). Both pathways work through differential regulation of gibberellin biosynthesis with *SVL* inducing *GA2 oxidase* in the developing bud, presumably in order to reduce the amount of active GAs that can reach the shoot apex, while, at the same time, repressing the expression of *GA20 oxidase* to downregulate GA biosynthesis. We show that *SVL* functions through a similar pathway in leaves. Grafting studies in *Populus* have shown that not only *FT2* but also *GA20 oxidase* expression in leaves, clearly affect growth cessation and bud set (Miskolczi et al., 2019), suggesting that both FT2 and GAs are moving from leaf to shoot apex in order to modulate this process. Our data show that SVL affects the expression of both *FT2* and *GA20 oxidase* in leaves and that SVL associates with both the *FT2* and *GA20 oxidase2* loci that both contain putative SVL binding sites in the form of CArG boxes shown to bind to Arabidopsis SVP (Gregis et al., 2013). However, in contrast to our findings here, Singh et al. (2019) could not detect an interaction between SVL and the *GA20 oxidase* loci. This discrepancy could be attributed to the use of different protocols and primers for the ChIP assay, but could also be related to the fact that we looked for interactions in leaf samples rather than in shoot apex samples as used by Singh et al. (2019). SVP-like proteins acts in complexes with other MADS-box proteins and co-transcription factors. These other factors are likely to differ between leaf and shoot apex samples and might affect the SVL binding. They might also contribute to an indirect binding to the *GA20 oxidase* locus in the ChIP assay.

One of the direct targets of *FT2* in the shoot apex is the gene *Like-AP1* (*LAP1*) which in turn mediates the regulation of cell cycle-related genes that are downregulated during growth cessation. (Azeez et al., 2014). Is it possible that *LAP1* is also a SVL target? Although our grafting experiments did not allow us to collect enough material to analyze the expression of target genes in the shoot apex, we did check for *LAP1* expression in leaves of *SVL* RNAi plants. *LAP1* is normally expressed to very low levels in leaves, but is dramatically upregulated in leaves of *SVL* RNAi trees (Supplementary Figure S7). The *LAP1* locus also contain several potential SVL binding sites (Supplementary Figure S7). Although the relevance of the *LAP1* regulation in the leaf is unclear, it shows a potential for SVL to also control growth cessation through a repression of *LAP1* expression in the shoot apex that could be part of the explanation to the contribution of SVL expression for growth cessation in both leaf and shoot apex (Figure 2D).

Our data suggest that SVL has only minor effects on the leaf transcriptome, suggesting that it might have relative few targets in the leaf compared to in the shoot apex. When the dataset was specifically quired both *FT2* and *GA20 oxidase2* were not found to be higher expressed in the SVL RNAi dataset compared to wild type after the shift to SD (not shown). The reason for these genes not appearing in the DEG list is probably due to relatively low expression levels and/or the fact that this was samples from a single time-point at the end of the day when expression was not significantly different.

**Figure 5:**
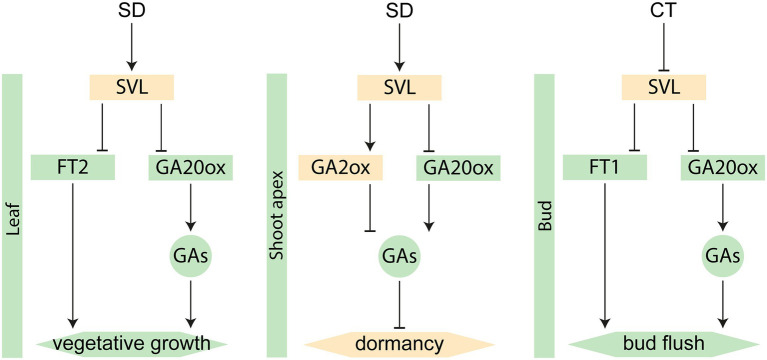
Different roles of SVL in the annual growth cycle. Green color indicates growth-promoting factors, while orange color indicates repressors. Boxes represent genes and circles represent hormones. SD = short day, CT = cold treatment.

Additionally, *SVL* has been shown to affect *FT1* expression in buds. Thus, *SVL* is involved in three similar pathways regulating different aspects of the annual growth cycle of *Populus* trees (Figure 5); First through a regulation of the photoperiod response in leaves, contributing to the downregulation of *FT2* and *GA20 oxidase*, leading to growth cessation and bud set (this study); Then as an inducer of *GA2 oxidase* and *CALLOSE SYNTHASE 1* in the buds to prevent growth-inducing signals to reach the shoot apex to establish dormancy (Singh et al., 2019); And finally, its expression is reduced in response to low temperatures in winter, leading to a relieved repression of *FT1* and reduced expression of *TCP18/BRC1*, hypothesized to lead to bud break (Singh et al., 2018). Consequently, SVL serves as an important regulator of both the beginning and end of the growing season as well as the establishment of winter dormancy. Interestingly, these three different phases of *SVL* regulation corresponds to three different clusters of expression profiles for *DAM* and *SVP*-like genes from different fruit tree species (Falavigna et al., 2019, 2021). In Rosaceae, *DAM* and *SVP*-like genes have evolved into different clades. In apple, the SVP-like protein SVPa provides DNA-binding activity to different complexes with DAM proteins that are specifically expressed during different phases of the dormancy cycle (Falavigna et al., 2021). Our data from growth cessation together with the previous data from bud set and bud break (Singh et al., 2018, 2019) suggest that this could also be true in *Populus* trees, with SVL serving as the central DNA-binding hub.

## Data Availability Statement

The datasets presented in this study can be found in online repositories. The names of the repository/repositories and accession number(s) can be found at: https://www.ebi.ac.uk/ena, PRJEB46749.

## Author Contributions

DA, JZ, and ON planned the research. DA, JZ, BZ, MR, KL, and AM performed experiments and analyzed data. DA and ON wrote the manuscript and all authors reviewed and approved its final version.

## Funding

VR supported PhD positions for AM and DA. Kempe foundation supported a post-doctoral stipend for JZ. VINNOVA and KAW supported positions, consumables, and platform support for BZ, MR, and DA. This work was supported by grants from the Swedish Research Council, the Knut and Alice Wallenberg Foundation, Kempe Foundation and the Swedish Governmental Agency for Innovation Systems (VINNOVA).

## Conflict of Interest

The authors declare that the research was conducted in the absence of any commercial or financial relationships that could be construed as a potential conflict of interest.

## Publisher’s Note

All claims expressed in this article are solely those of the authors and do not necessarily represent those of their affiliated organizations, or those of the publisher, the editors and the reviewers. Any product that may be evaluated in this article, or claim that may be made by its manufacturer, is not guaranteed or endorsed by the publisher.

## Footnotes

^1^ http://www.bioinformatics.babraham.ac.uk/projects/fastqc/

^2^ https://www.ebi.ac.uk/ena/
