# Supplementary material for: Populus SVL Acts in Leaves to Modulate the Timing of Growth Cessation and Bud Set
Source: Front Plant Sci. 2022 Feb 17;13:823019. doi: 10.3389/fpls.2022.823019 (PMC8891642; doi:10.3389/fpls.2022.823019)
Supplement: Supplementary file 1 [file Data_Sheet_1.PDF]

Table S1: List of used primers

**ChIP**	
PtraGA20ox_P1f	AAGGGAGATTGAAGCATGTT
PtraGA20ox_P1r	CCTATGATCTCCCTCTCTAC
PtraGA20ox_P2f	TCGGAAGTTGGAGAAACCT
PtraGA20ox_P2r	TAATTCAAGCCATGGGAGT
PtraGA20ox_P3f	CTCGGCATCATCACCTATAT
PtraGA20ox_P3r	CTGGTTTTGTTGATTGTGGT
PtraGA20ox_P4f	TCAACAAAACCAGTCCCAAA
PtraGA20ox_P4r	GGGCAGGGACATTTTATTCT
PtraGA20ox_P5f	TGAAAAGCAACCGCAATGAT
PtraGA20ox_P5r	TCCATATACCGAATGCCTAA
PtraGA20ox_P6f	GTCCCTCAAAACAGATTTCT
PtraGA20ox_P6r	AACCCATAACTTCCTGTTCT
FT2chipF1	CCATATATCTTCGAGCGTTGCA
FT2chipR1	AAGCTGGGTTCGAGTAAAG
FT2chipF2	GTATGCCGAGATGGAGACT
FT2chipR2	TCATAAAGCATGCATGGACC
FT2chipF3	GATTCGTAAGTGTACACTCG
FT2chipR3	CTCACCATAATAGTCCTATC
FT2chipF4	GGAAAACGTGAATCTGGCTC
FT2chipR4	GGTGATGCCTCGAGGCTCA
FT2chipF5	ATGCCTAGGGATAGAGACCCT
FT2chipR5	AGAAGGTCCTTAGATCTTC
FT2chipF6	GGTGATGGTGAGTCCTTGGA
FT2chipR6	CGTCGACGTACAGGTGAAGT
**qPCR**	
18SF	TCAACTTTCGATGGTAGGATAGAG
18SR	CCGTGTCAGGATTGGGTAATTT
UBQF	GTTGATTTTTGCTGGGAAGC
UBQR	GATCTTGGCCTTCACGTTGT
FT2F	AGCCCAAGGCCTACAGCAGGAA
FT2R	GGGAATCTTTCTCTCATGAT
SVLF	ATGAGAGACTCAAACAGCAAGTGG
SVLR	ACTGCCCTTCCTCGTAACCAAC
GA20oxF	GGTGACACCTTCATGGCTCTATCG
GA20oxR	GTGTTTGGCTGTTCACCACTG
**Cloning**	
SVLoeF	ATGGCAAGAGAGAGGATTCAGA
SVLoeR	TCAAACAGCAGACAAACACA
SVLRNAiF	GGGGACAAGTTTGTACAAAAAAGCAGGCTAGGCAAATGAGAGGGGAAGA
SVLRNAiR	GGGGACCACTTTGTACAAGAAAGCTGGGTCGGCCATTAGATATCTCCAC

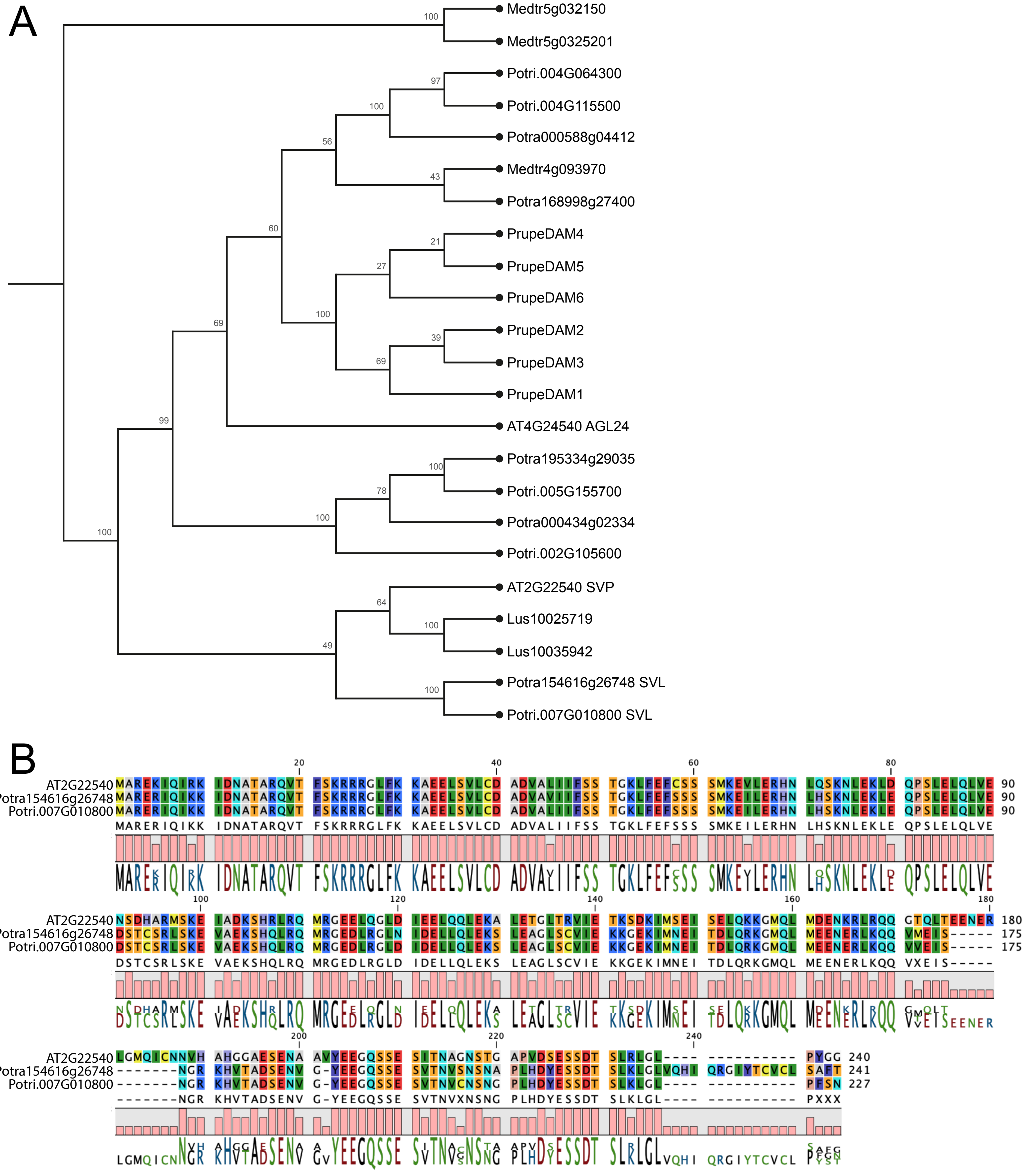

Figure S1:

A) Phylogenetic tree of MADS box containing proteins of *Populus trichocarpa* (Potri), *Populus tremula* (Potra), *Arabidopsis thaliana* (AT), *Medicago truncatula* (Medtr), *Prunus persica* (Prupe) and *Linum usitatissimum* (Lus).

B) SVL protein alignment of *Arabidopsis thaliana*, *Populus trichocarpa* and *Populus tremula*.

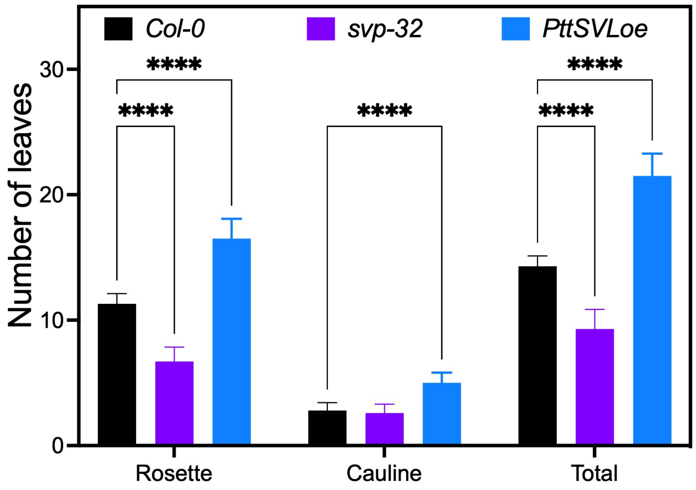

Figure S2:
Populus SVL is functionally conserved with Arabidopsis SVP and can rescue the svp-32 mutant phenotypes. Rosette and cauline eaves of 10 plants per genotype were counted until the formation of the first flowers to determine flowering time. The asterisks indicate a statistically significant difference of each genotype from WT by Welch's test. Error bars indicate standard error, n = 10. **** indicate $p < 0.0001$

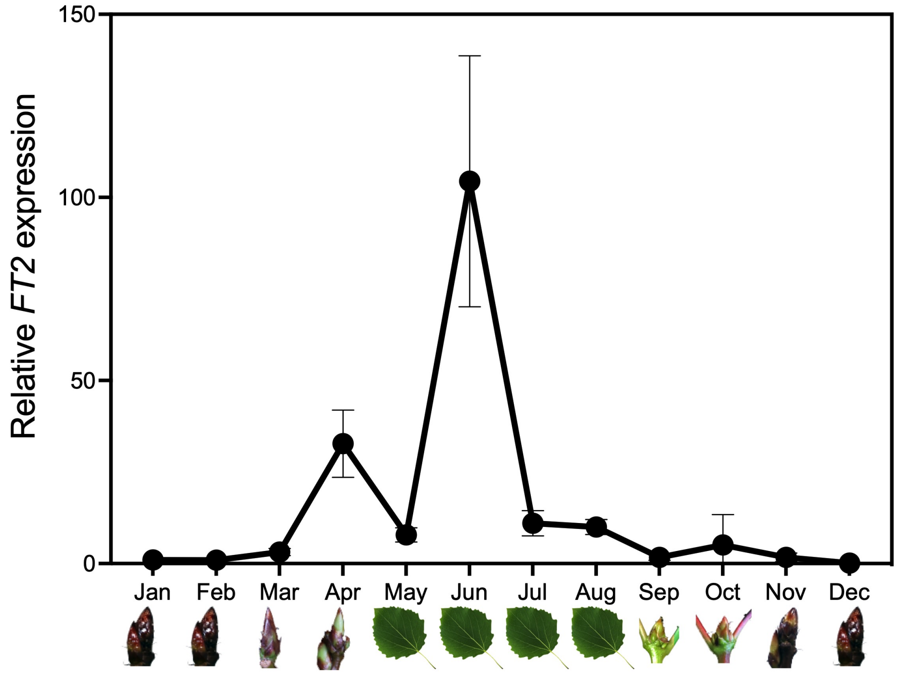

Figure S3:
Expression of *FT2* in field-grown mature *Populus tremula* over the course of one year. Samples were taken at 2 pm in the middle of each month. May-August leaves, September-April terminal buds. Error bars indicate standard error, n =3.

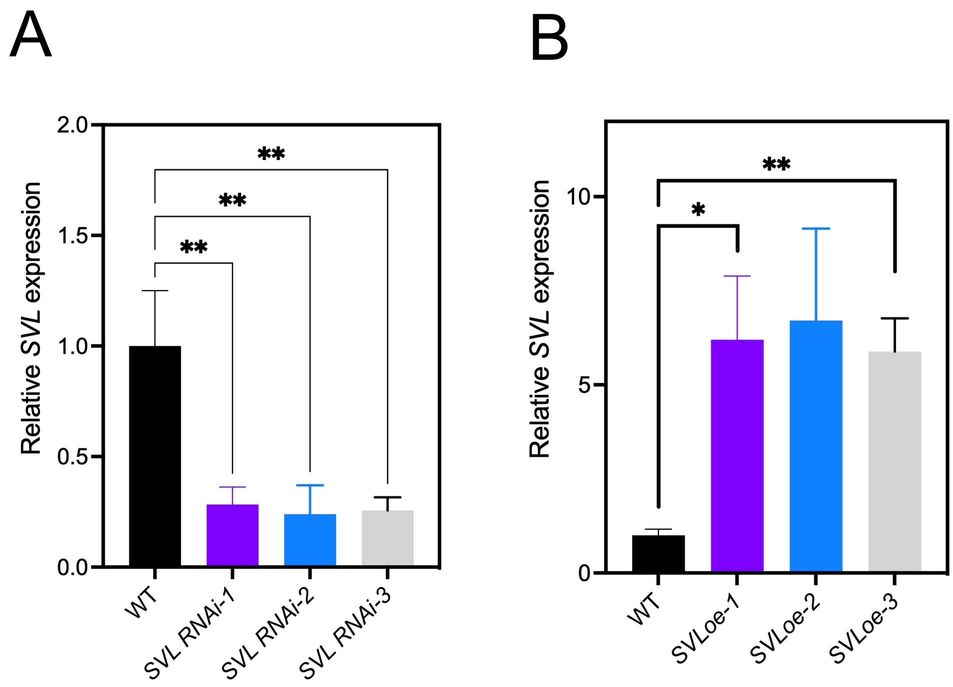

Figure S4:

A) SVL expression is reduced to less that 30% in *SVL* RNAi lines.Samples were taken at ZT4.
B) SVL expression is overexpressed in *SVLoe* lines. Samples were taken at ZT17.
The asterisks indicate a statistically significant difference of each line from WT by Welch's test.
Error bars indicate standard error, n = 3. * indicate $p < 0.05$, ** indicate $p < 0.01$.

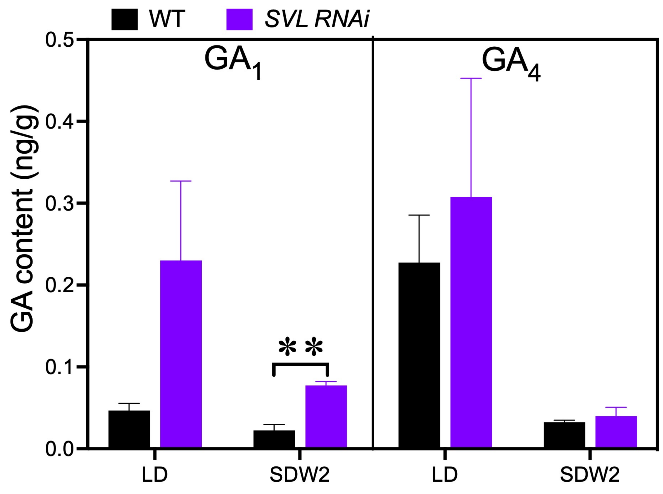

Figure S5:
GA_1 and GA_4 content in leaves of WT and *SVL* RNAi line in LD and after two weeks of SD treatment. The asterisks indicate a statistically significant difference of *SVL* RNAi line from WT by Welch's test. Error bars indicate standard error, n = 4. ** indicate $p < 0.01$.

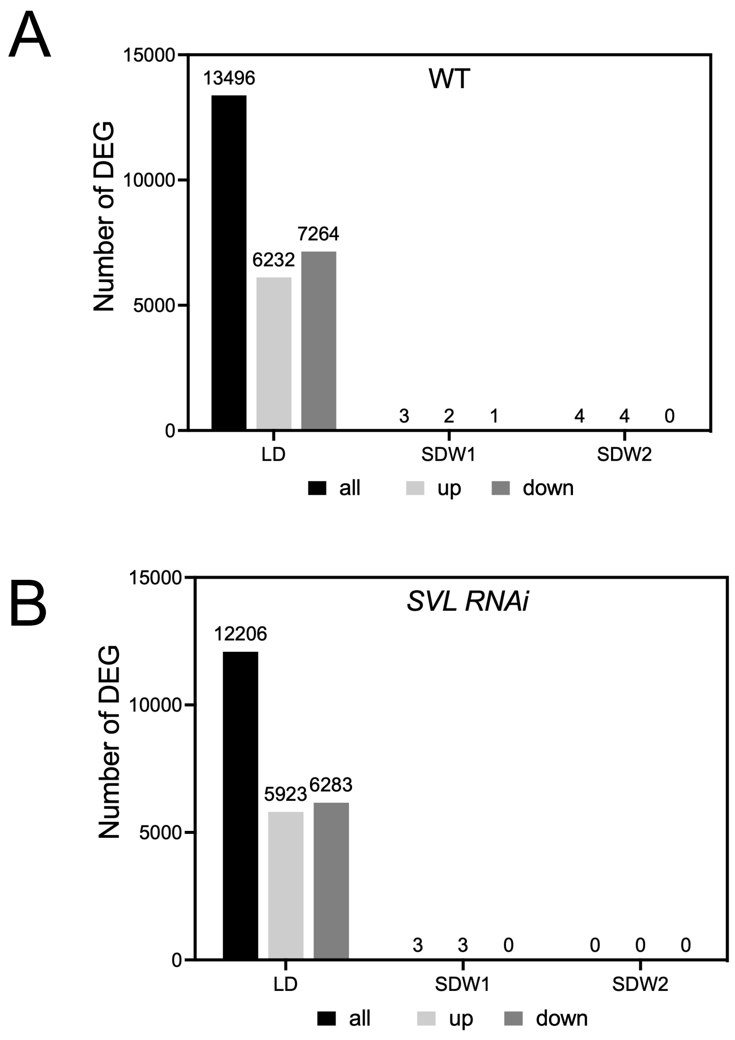

Figure S6:
A) Number of differentially expressed genes (DEG) in WT over the time course of the experiment.
B) Number of differentially expressed genes (DEG) in *SVL* RNAi over the time course of the experiment.

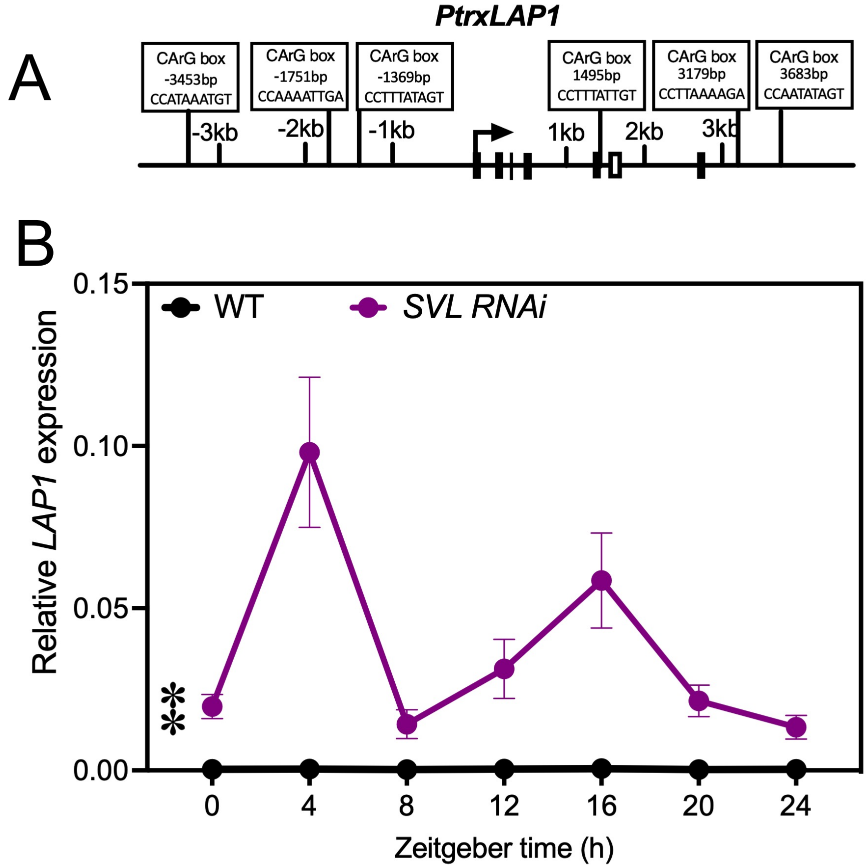

Figure S7:

A) Genomic structure of *PtrxLAP1*. Six potential SVL-binding sites, CArG boxes (NC[A/T]6GN) shown to bind to Arabidopsis SVP (Gregis et al., 2013), are indicated.
B) Gene expression of *LAP1* after two weeks of SD (14h light/ 10h dark) treatment. The asterisks indicate a statistically significant difference between SVL RNAi and WT samples by two-way ANOVA Fisher's test. Error bars indicate standard error, n = 3. ** indicate $p < 0.01$
